# The Effects of 2′,4′-Dihydroxy-6′-methoxy-3′,5′- dimethylchalcone from *Cleistocalyx operculatus* Buds on Human Pancreatic Cancer Cell Lines

**DOI:** 10.3390/molecules24142538

**Published:** 2019-07-11

**Authors:** Huynh Nhu Tuan, Bui Hoang Minh, Phuong Thao Tran, Jeong Hyung Lee, Ha Van Oanh, Quynh Mai Thi Ngo, Yen Nhi Nguyen, Pham Thi Kim Lien, Manh Hung Tran

**Affiliations:** 1 Hanoi University of Pharmacy, 13 Le Thanh Tong Street, Hoan Kiem District, Hanoi 100100, Vietnam; 2 Faculty of Pharmacy, Nguyen Tat Thanh University, 300C Nguyen Tat Thanh Street, District 4, Hochiminh City 72820, Vietnam; 3 Department of Biochemistry, College of Natural Sciences, Kangwon National University, Chuncheon, Gangwon-Do 24414, Korea; 4 College of Pharmacy, Hai Phong University of Medicine and Pharmacy, 72A Nguyen Binh Khiem, Hai Phong 180000, Vietnam; 5 Faculty of Biology and Biotechnology, University of Science, Vietnam National University Hochiminh City, 227 Nguyen Van Cu, District 5, Hochiminh City 748000, Vietnam; 6 Biomedical Sciences Department, Institute for Research & Executive Education (VNUK), The University of Danang, 158A Le Loi, Hai Chau District, Danang City 551000, Vietnam

**Keywords:** *Cleistocalyx operculatus*, 2′,4′-dihydroxy-6′-methoxy-3′,5′-dimethylchalcone (DMC), pPancreatic cancer, PANC-1

## Abstract

2′,4′-Dihydroxy-6’-methoxy-3′,5′-dimethylchalcone (DMC), a principal natural chalcone of *Cleistocalyx operculatus* buds, suppresses the growth of many types of cancer cells. However, the effects of this compound on pancreatic cancer cells have not been evaluated. In our experiments, we explored the effects of this chalcone on two human pancreatic cancer cell lines. A cell proliferation assay revealed that DMC exhibited concentration-dependent cytotoxicity against PANC-1 and MIA PACA2 cells, with IC_50_ values of 10.5 ± 0.8 and 12.2 ± 0.9 µM, respectively. Treatment of DMC led to the apoptosis of PANC-1 by caspase-3 activation as revealed by annexin-V/propidium iodide double-staining. Western blotting indicated that DMC induced proteolytic activation of caspase-3 and -9, degradation of caspase-3 substrate proteins (including poly[ADP-ribose] polymerase [PARP]), augmented bak protein level, while attenuating the expression of bcl-2 in PANC-1 cells. Taken together, our results provide experimental evidence to support that DMC may serve as a useful chemotherapeutic agent for control of human pancreatic cancer cells.

## 1. Introduction

Pancreatic cancer (PC) causes significant mortality in the USA and other countries [1]. The GLOBOCAN 2012 summit reported that PC is responsible for over 331,000 deaths annually, and is the seventh leading cause of cancer deaths in both males and females [2,3]. PC includes adenocarcinomas, accounting for approximately 85% of cases, with an overall 5-year survival rate of 5–10%, and endocrine tumors constituting less than 5% of all cases [2,3]. The causes remain insufficiently known; however, previous studies have established that the risk factors include obesity, a genetic predisposition, diabetes, a poor diet, and physical inactivity. In addition, smoking was recognized to be a risk factor of PC. [4,5]. Over the past 10 years, PC mortality has increased in both genders in the USA, Europe, Japan, and China [4,5]. Currently, there are no effective screening recommendations for PC; therefore a better understanding of the cause and identification of risk factors is essential to prevent this disease [6]. Several therapies for PC such as radiotherapy, chemotherapy, and immunotherapy have been developed; however, drug development for this cancer remains challenging. In the search for new anti-PC drugs, natural products have been identified as potential sources for the development of new drugs [7,8].

**Figure 1:**
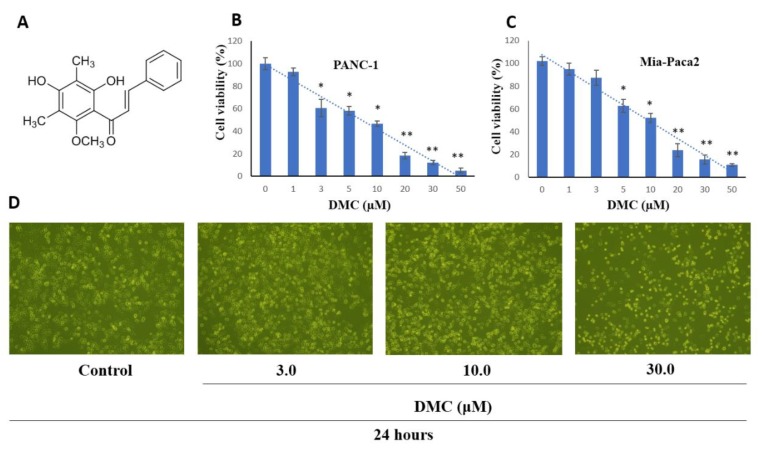
(**A**) Chemical structure of DMC; Effect of DMC on PANC-1 (**B**), and MIA-PACA2 (**C**) cell viability; and (**D**) PANC-1 cell morphology visualized by light microscopy (scale bar 500 µm), cells were seeded into 6-well plates at 1 × 10^5^ cells/well and treated with the indicated concentration of DMC for 24 h. Data are presented as the mean ± standard deviation of three independent experiments performed in duplicate (*p < 0.01; **p < 0.05).

*Cleistocalyx operculatus*, a member of Myrtaceae family, had been used as a beverage since ancient times in Vietnam for the treatment of cold, fever, inflammation, and gastrointestinal disorders [9]. A bud water extract increased contractility and decreased the frequency of contraction in an isolated rat heart perfusion system. Moreover, data from several studies also suggested that this extract protected lipid peroxidation in rat liver microsomes and the trauma of PC12 cells; and inhibited α-glucosidase, rat-intestinal maltase, and sucrase activities [10,11,12,13]. The plant contains chalcones, flavanones, flavones, and triterpenoids exhibiting many pharmaceutical activities, including anti-tumor effects; inhibition of cancer cell growth; and anti-cholinesterase, anti-oxidation, anti-hyperglycemia, anti-influenza, and anti-inflammation activities [14,15,16,17,18]. Of the active compounds, the chalcone 2′,4′-dihydroxy-6′-methoxy-3′,5′-dimethylchalcone (DMC) exhibited both cytotoxic and anti-tumor effects in vivo and was cytotoxic to several cancer cell lines in vitro. DMC could reverse multi-drug resistance in HCC cell lines. Moreover, this compound displayed hepatoprotection and neuroprotection, promoted glucose uptake, affected the differentiation of 3T3-L1 cells into adipocytes; and reduced drug efflux by suppressing Nrf2/ARE signaling in human HCC BEL-7402/5-FU cells [19,20,21,22]. DMC triggers SMMC-7721 cell apoptosis via the mitochondrion-dependent pathway, inhibiting Bcl-2 expression and thus causing outer mitochondrial membrane disintegration [23]. DMC is the most cytotoxic agent isolated from the plant to date. Here, we isolated DMC (Figure 1A) from buds of *C*. operculatus using several chromatographic steps, and explored the effects thereof on some human cancer cell lines. We also provide the first evidence that DMC induces apoptosis of the human pancreatic cancer cell lines PANC-1.

## 2. Results

### 2.1. Cell Proliferation Activity

To investigate the effects of DMC on the human pancreatic cancer cell lines PANC-1 and MIA PACA2 growth, cells were treated with DMC (3–30 μM) for 48 h, and after that, cell numbers and viability were measured using a Dojindo kit. DMC significantly inhibited PANC-1 (Figure 1B) and MIA PACA2 cell proliferation (Figure 1C) in concentration-dependent manners, with IC_50_ values of 10.5 ± 0.8 and 12.2 ± 0.9 μM, respectively. Inverted microscopy revealed that exposure to DMC for 24 h greatly affected the number of cell death of PANC-1 cells (Figure 1D). Considering that DMC showed stronger toxicity against PANC-1 than MIA PACA2 our subsequent studies focused on the mechanism of action of DMC in PANC-1.

### 2.2. Caspase-3 activity

Caspase-3 is a member of the cysteine-aspartic acid protease family and usually exists as an inactive precursor of 32 kDa in size. When it is in activation mode, this causes the death of cell by an apoptosis pathway via cleavage of proteins into heterozygous substances. DMC (3–30 µM) was added to PANC-1 cells (1 × 10^6^/well) followed by incubation for 12, 24, and 48 h; this enhanced caspase-3 activation was measured by assaying the levels of Ac-Asp-Glu-Val-Asp-8- amino-4-trifluoromethylcoumarin (Av-DEVD-AFC).

**Figure 2:**
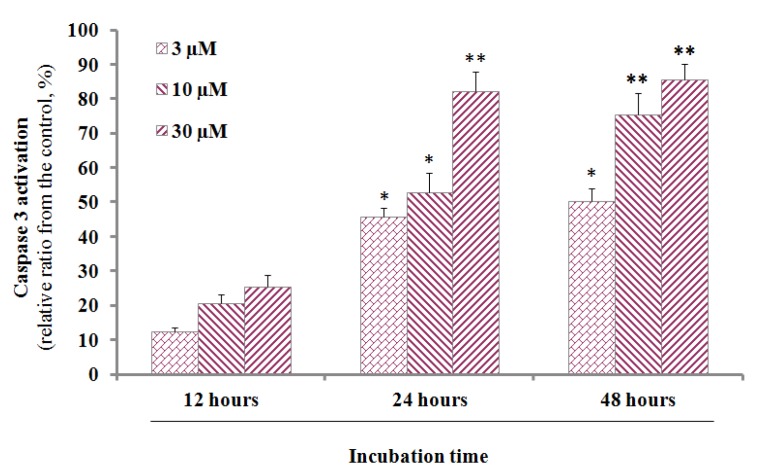
The increment of caspase-3 activity in PANC-1 cells treated by DMC in vitro. After 12 h, 24 h and 48 h incubation with DMC (3–30 μM), the cell lysates were incubated at 37 °C with caspase-3 substrate (Ac-DEVD-AFC) for 1 h. The fluorescence intensity of the cell lysates was measured to determine the caspase-3 activity. The blank group was used as 0.1% DMSO-treated cells. Data are presented as the mean ± SD of results from three independent experiments (* *p* < 0.01; ** *p* < 0.05).

Figure 2 shows that caspase-3 activity increased 3–9-fold in a dose-dependent manner, when DMC-induced activities were compared to those of the vehicle.

### 2.3. Induction of Apoptosis by DMC

**Figure 3:**
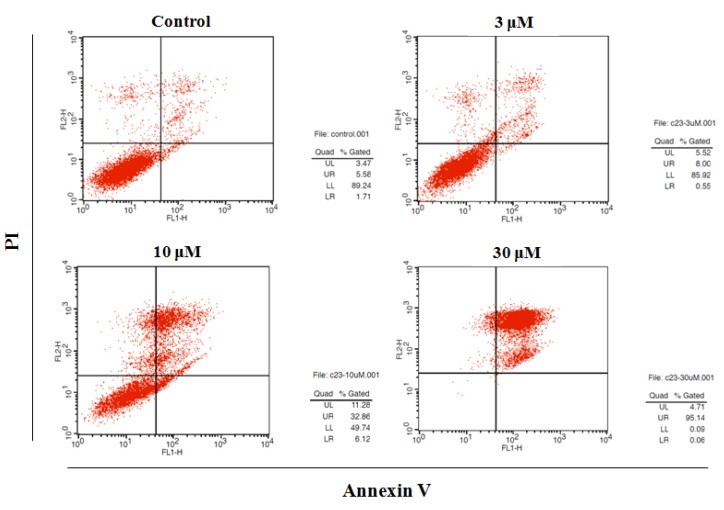
Effect of DMC on apoptosis of PANC-1 cells. Apoptosis quantification using annexin V/PI double staining assay after treatment with DMC (3–30 μM) for 48 h. PANC-1 cells were harvested and stained with PI and annexin V-FITC in darkness for 15 min. Data are presented as the mean ± SD of results from three independent experiments.

Next, PANC-1 cells (5 × 10^5^) were treated with DMC (3–30 μM) for 48 h, stained with annexin V/PI, and subjected to flow cytometry using a BD Biosciences platform. Early and late apoptotic cells, and necrotic cells, were counted; and total and early apoptosis quantified (Figure 3). Apoptotic cell numbers increased in a DMC dose-dependent manner.

### 2.4. Effect of DMC on the Expression of Apoptosis-Related Protein

**Figure 4:**
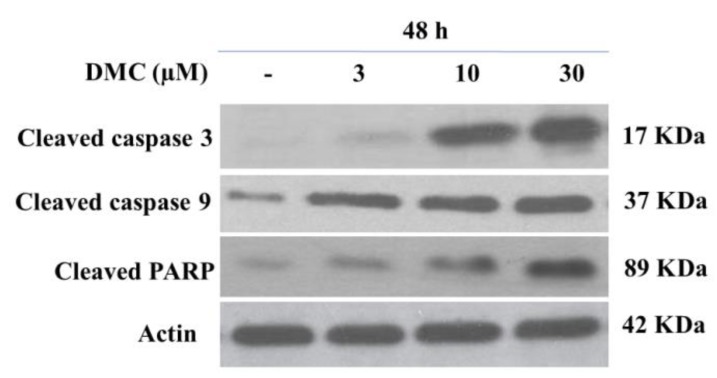
Effect of DMC on caspase activation and PARP degradation protein expression in PANC-1 cells. Cells were treated with DMC (3–30 µM) for 48 h. Protein 50 µg/lane from cells lysates were electrophoresed on SDS-PAGE gels, then transferred to total blot PVDF membranes. β-Actin was used as a control, (–), 0.1% DMSO-treated cells. The experiments were carried out in three replicates.

**Figure 5:**
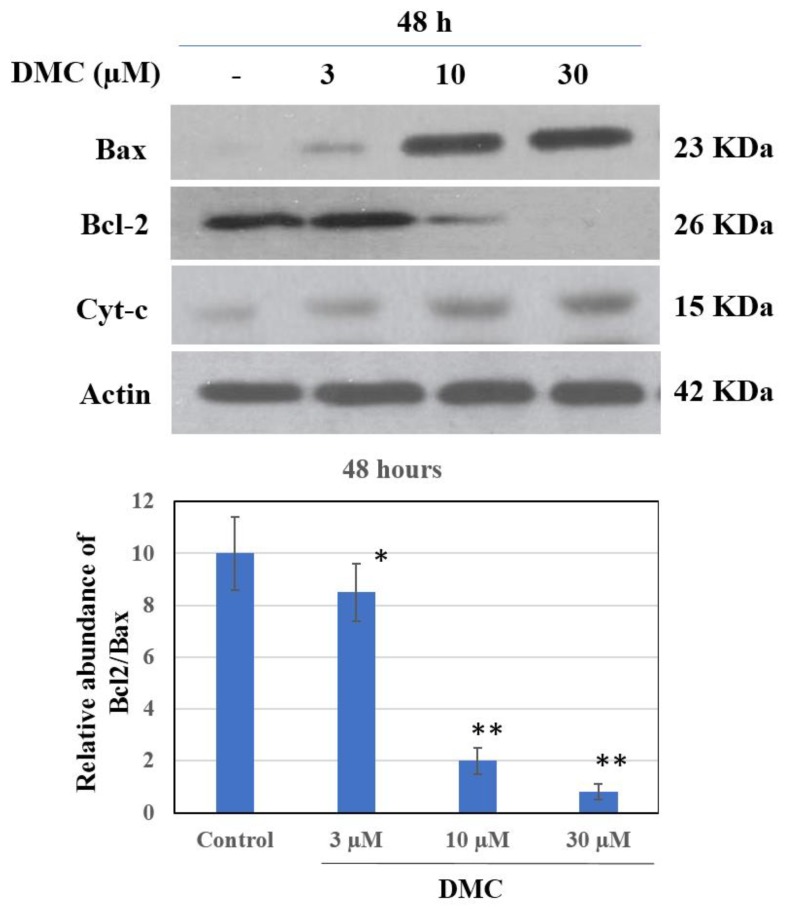
Effect of DMC on Bcl-2, Bax and Cyt-c protein expression in PANC-1 cells. Cells were treated with DMC (3–30 µM) for 48 h. Protein 50 µg/lane from cells lysates were electrophoresed on SDS-PAGE gels, then transferred to total blot PVDF membranes. β-Actin was used as a control, (–) 0.1% DMSO-treated cells. The experiments were carried out in three replicates. * *P* < 0.05 and *P* < 0.01 compared with control group.

As the apoptotic cell population thus increased dramatically, we next measured the levels of apoptotic proteins. We used western blotting to detect death receptors and pro-apoptotic ligands that might be involved in DMC (3–30 µM)-induced PANC-1 apoptosis. As shown in Figure 4, DMC significantly inhibited expression of the anti-apoptotic Bcl-2 protein in a dose-dependent manner. Notably, the levels of the pro-apoptotic Bax protein were also changed by DMC. Recent evidences have suggested that the mitochondrial mutilation expedited cytochrome c (Cyt-c) which was discharged from mitochondria into the cytoplasm, triggering apoptotic progression. This process caused the stimulation of the caspase signaling and mitochondria-facilitated apoptosis so we assessed whether DMC triggers apoptosis via this mechanism in PANC-1 cells. We used western blotting to measure Cyt-c protein levels. DMC upregulated cytosolic Cyt-c expression and downregulated Bcl-2 synthesis compared to untreated cells (Figure 5). One of the other substrates for caspase during apoptosis is PARP, an enzyme that appears to be involved in DNA repair and genome surveillance and integrity in response to environmental stress. The beginning of caspase signaling activation might cause PARP cleavage which was considered as the main pathway in triggering apoptosis. As shown in Figure 4, exposure to DMC (3–30 μM) for 48 h triggered progressive PARP proteolytic cleavage and/or downregulation. We used western blotting to quantitate the levels of cleaved caspase-3 and -9; DMC upregulated cleavage of both proteins (Figure 4), explaining the Bcl-2 downregulation evident in Figure 5.

## 3. Discussion

The pear-shaped pancreas—an abdominal organ located horizontally behind the lower part of the stomach—is an important component of the digestive system, secreting hormones, including insulin, that regulate sugar metabolism and digestive enzymes. Pancreatic cancer begins in the tissues of the pancreas. This cancer usually has a poor prognosis, even when the patient is diagnosed at the early stage because its signs and symptoms are hard to identify. The symptoms of pancreatic cancer generally mostly appear at the advanced stages of the disease. Some of the signs and symptoms of pancreatic cancer patients that might be identified include upper abdominal pain that spreads to the back, jaundice, yellow eyes, loss of appetite, weight loss, and depression. At present, the causes of the cancer remain unclear. There are two types of pancreatic cancer, including cancer formed in the pancreas (adenocarcinoma) and cancer formed in hormone-producing cells which is called endocrine. Pancreatic cancer is one of the most prevalent malignant tumors in the world and the treatment regimens for pancreatic cancer primarily depend on the cancer stages [7,8]. Nowadays, about 15–20% of patients undergo surgery and only 5% of them survive to 5 years [8]. The recent increase in cancer incidence, the absence of a cure, and severe side effects of existing drugs render it essential to find new effective therapeutics. In Vietnam, both Western and Oriental (natural plant) medicines are used to treat pancreatic cancer. Oriental medicines have fewer side effects and are less expensive than Western drugs. The herbal remedies used also target cancer-related impacts on the spleen, and sputum production. The recommended medicines include Radix Astragali membranacei, *Scutellaria barbata*, *Plumbago zeylanica*, *Poria cocos*, *Angelica sinensis*, and Rhizoma atrclylodis macrocephalae [9]. However, one of the limitations in the treatment of this disease by traditional oriental medicine method is due to the lack of scientific research perspectives in using of medicinal herbs with different ingredients and amounts.

In the last 10 years, many natural medicinal products have been used to treat pancreatic cancer. Fucoidan from a seaweed collected in Okinawa destroyed pancreatic tumor cells, and tumors regressed after 4–5 years of treatment [24]. α-Bisabolol (a sesquiterpene essential oil ingredient) reduced proliferation and survival of the pancreatic cancer cell lines KLM1, KP4, Panc1, and MIA Paca2; but not a pancreatic epithelial cell line (ACBRI515) [25]. Daily intake of plants rich in flavonoids and proanthocyanidins reduces the risk of pancreatic cancer by 25% [26,27,28]. Ethyl acetate extracts of *Coreopsis tinctoria* rich in flavonoids such as marein and flavanomarein kill pancreatic tumor cells by inducing apoptosis [29]. *Scutellaria baicalensis* extracts containing baicalein, wogonin, oroxylin A, and a glucuronide effectively countered pancreatic cancer in a mouse xenograft model [30,31]. The natural flavonoids and chalcons have many pharmaceutical applications, including antioxidant and anticancer ones. 2′,4′-Dihydroxy-6′-methoxy-3’,5’-dimethylchalcone (DMC) is also an important natural chalcone that has been shown to exhibit tremendous pharmacological activities which include anticancer activity against the wide range of cancer types. However, the anti-pancreatic cancer activity of DMC has not been previously investigated. In this study, DMC was selected to investigate the capability against PANC-1 cell lines. To clarify mechanism responsible for its anticancer activity, DMC at the concentration of 3–30 µM enhanced annexin-V uptake in PANC-1 cells signifying traslocation of the cell membrane phospholpids, phosphatidylsenin, from inner face to the outer surface of plasma membrane of PANC-1 cells then led to the cell apoptosis (Figure 3). Apoptosis, however, is known to be triggered by different routes, and the mitochondrials enhancement is a popularly crucial signalling pathway in the induction of apoptosis progress. Among these mitochindrials, the Bcl-2 family proteins are frequently main factors in apoptotic pathway due to their natural functional property. Bcl-2 family proteins play major roles in apoptosis and it has been suggested that such proteins exert either pro- or anti-apoptotic effects. The proteins either activate or inactivate transport through inner mitochondrial membrane pores, thus regulating the matrix Ca^2+^ level, the pH, and the cell membrane potential. Some pro-apoptotic Bcl-2 proteins may induce cytochrome c (Cyt-c) release to the cytosol; anti-apoptotic Bcl-2 proteins may inhibit such release. Cytosolic Bcl-2 proteins activated caspase-9 and -3, triggering apoptosis, meanwhile Bax was once termed Bcl-2-like protein 4, and is a pro-apoptotic protein; Bcl-2 is a major anti-apoptotic protein. Bax in the outer mitochondrial membrane enables the release of Cyt-c and activates caspase-9, a cysteine-aspartic protease involved in apoptosis and cytokine signaling. Caspase-3 is activated by proteolytic cleavage of caspase 9 to play a key role in apoptosis, further stimulating Cyt-c release by mitochondria and activating apaf-1 (the apoptosome), which then cleaves the caspase-9 pro-enzyme to the active dimer. In human cancer cells, this enzyme is regulated via phosphorylation mediated by an allosteric inhibitor, inhibiting dimerization and inducing a conformational change. Stimulation of caspase signaling and the accompanying cleavage of PARP are the principal features of the apoptotic cascade. We found that DMC activated enzymes and the PARP pathway to induce PANC-1 cell death, augmented by changes in Bcl-2 and Bax expression levels. DMC triggered the dose-dependent release of Cyt-c from mitochondria into the cytoplasm of PANC-1 cells.

DMC induces apoptosis in several human cancer cell lines including SMMC-7721 (human hepatocarcinoma cancer cells), 8898 (pancreas cancer cells), HeLa (cervical cancer cells), SPC-A-1 (lung cancer cells), 95-D (metastatic lung carcinoma cells), and GBC-SD (gall bladder carcinoma cells). When SMMC-7721 cells were treated with DMC for 48 h, the DNA became fragmented and the chromatin condensed. Also, the proportion of hypodiploid SMMC-7721 cells increased after DMC treatment [32]. At a low concentration, DMC inhibited proliferation of the human leukemia cell line K562. Notably, DMC downregulated Bcl-2 protein expression but did not affect Bax protein expression, thus reducing the Bcl-2:Bax ratio [33]. DMC was not toxic to normal human liver L-02 or normal human fetal lung fibroblast HFL-1 cell lines. In SMMC-7721 cells, DMC induced apoptosis by increasing intracellular ROS generation via inhibition of N-acetylcysteine activity [23]. Our data partly explained why DMC triggers PANC-1 cell apoptosis. We conclude that DMC exhibits significant anti-PANC-1 cancer cell activity; however, further in vivo evaluation in a mouse model of pancreatic cancer is essential.

## 4. Material and Methods

### 4.1. General Experimental Procedures

NMR experiments were conducted on a Unity INOVA 400 spectrometer (Varian, IL, USA). ^1^H- and ^13^C-NMR spectra were recorded at 400 and 100 MHz, respectively, and tetramethylsilane was used as the internal standard. ESI MS analyses were performed on a Micromass QTQF2 mass spectrometer (Water, Milford, MA, USA). The untraviloet (UV) was measured with a Shimadzu UV-1800 UV-Vis spectrophotometer (Shimadzu, Japan). IR spectrum was measured with a Shimadzu IR-408 spectrophotometer in CHCl_3_ solution (Shimadzu, Japan). TLC was carried out on silica gel F_254_-precoated glass plates and RP-18 F_254S_ plates (Merck, Germany). Dulbecco’s modified Eagle medium (DMEM), fetal bovine serum (FBS), trypsin-EDTA 0.25%, streptomycin and penicillin were obtained from Hyclone (Logan, UT, USA). Dimethyl sulfoxide (DMSO), and a Dojindo Kit was purchased from Dojindo Molecular Technology INC (Maryland, USA). Annexin V-FITC/PI double staining detection kit, mitochondrial membrane potential assay kit with caspase-3 activity assay kit, propidium iodide (PI) were purchased from Beyotime (Beyotime Institute of Biotechnology, Shanghai, China). All used chemicals and reagents were of analytical grade.

### 4.2. Plant Material

The buds of *Cleistocalyx operculatus* were collected at Quang Nam province, Vietnam, in July 2017 and identified by Dr Pham Cong Tuan, Danang Traditional Medicine Hospital (Danang city, Vietnam). A voucher specimen (TMH-22-2017) was deposited in the Pharmaceutical Biology Laboratory of the University of Danang (Danang city, Vietnam).

### 4.3. Isolation of DCM

The air-dried buds (2.0 kg) were extracted with 70% ethanol (2 liters × 3 times). The 70% EtOH extract was combined and concentrated in vacuo to yield a residue which was suspended in water and then successively partitioned with *n-*hexane, EtOAc, and *n-*BuOH. After removal of solvent *in vacuo*, the *n-*hexane fraction was obtained (16.8 g). The *n-*hexane soluble fraction (HEX) was separated by silica gel column chromatography using a gradient of *n-*hexane−EtOAc (from 40:1 to 1:1) to yield 20 fractions (HEX.1 ~ HEX.20) according to their TLC profiles. Fraction HEX-5 (5.62 g) was further fractionated on a Sephadex LH-20 column eluting with MeOH to divide to six sub-fractions (HEX.5.1-HEX.5.6). 2’,4’-Dihydroxy-6’-methoxy-3’,5’-dimethylchalcone (DMC, 1.6 g) was obtained from HEX.5.2 by crystallization from MeOH.

*2′,4′-Dihydroxy-6′-methoxy-3’,5’-dimethylchalcone (DMC):* Orange yellow needles (MeOH), mp 124–125 °C, UV *λ*_max_ (MeOH) nm (log ε): 284 (4.15), 320 (4.13); IR (KBr) cm^−1^: 3460, 2875, 2750, 1630, 1550, 1450; ESI-MS *m*/*z* 312.1 [M]^+^ (calcd for C_18_H_16_O_5_), for ^1^H and ^13^C-NMR spectral data please see Supplementary Materials and in the comparison with previous reference [10].

### 4.4. Cell Lines and Culture

The human pancreatic cancer cell lines PANC-1 (human pancreas) and MIA-PACA2 (human pancreatic carcinoma) were obtained from the American Type Culture Collection (ATCC, Manassas, VA, USA). The cells were maintained in DMEM (GibcoBRL, NY, USA) with 10% fetal bovine serum (FBS) supplemented with 2% penicillin and 100 μg/mL of streptomycin at 37 °C in a 95% humidified atmosphere containing 5% CO_2_.

### 4.5. Cell Proliferation Activity Assay

Cell proliferation activity of DMC was determined against PANC-1 and MIA-PACA2 cancer cell lines using a Dojindo kit with a slight modification. Viable cells were seeded in the growth medium into 96-well microtiter plates (95 μL, concentration 1 × 10^4^ cells/well) and incubated at 37 °C in a 5% CO_2_ incubator. The test sample DCM was dissolved in DMSO and adjusted to final sample concentrations ranging from 3 to 30 μM by diluting with the growth medium. Each sample was prepared in triplicate. The final DMSO concentration was adjusted to <0.1%. After standing for 4 h, the test sample was added to each well. The same volume of medium with 0.1% DMSO was added to the control wells. After 48 h incubation, Dojindo reagent was added to the each well (10 μL). 4 h later, the plate was removed from incubator and the optical density (O.D) was measured at 450 nm using a Molecular Devices microplate reader (Molecular Devices, Sunnyvale, CA, USA). The IC_50_ value was defined as the concentration of sample which reduced absorbance by 50% relative to the vehicle-treated control.

### 4.6. Caspase-3 Actyivation Assay

Caspase-3 enzyme activity was measured by proteolytic cleavage of the fluorogenic substrate Ac-DEVD-AFC by counting on a fluorescence plate reader (Twinkle LB970 microplate fluorometer, Berthold Technologies, Bad Wildbad, Germany). PANC-1 cells (1 × 10^5^ cell/well) were treated with DCM (3–30 μM). After incubation for 24 h, cells were harvested and washed with cold PBS. The pellets were lyzed using 15 μL of lysis buffer [10 mM Tris-HCL (pH 8.0), 10 mM EDTA, 0.5% Triton X-100] at room temperature for 10 min, and then placed on ice; 100 μL of assay buffer [100 mM Hepes (pH 7.5), 10 mM dithiothreitol, 10% (*w*/*v*) sucrose, 0.1% (*v*/*v*) Chaps, 0.1% (*v*/*v*) BSA] and 10 μL of substrate solutin (200 μm substrate in assay buffer) were added. After incubation at 37 °C for 1 h, fluorescence was measured with excitation at 370 nm and emission at 505 nm.

### 4.7. Detection of Apoptosis by Double Stanning

The Annexin V-FITC/PI staining kit was used to detect the phosphatidylserine translocation, an important characteristic at an early stage of cell apoptosis. Briefly, PANC-1 cells were seeded in 6 well plates at a density of 2 × 10^5^ cells/mL and incubated for 24 h. After that, cells were treated with different concentrations of DMC for 48 h. The cells were collected and washed in PBS, then were resuspended in 195 μL binding buffer, and incubated with 10 μL Annexin V-FITC and 5 μL PI in the dark for 20 min. Thereafter, the solutions were immediately measured by FCM (Beckman, Fullerton, CA, USA).

### 4.8. Preparation of Total Cell Extract and Immuno Blot Analysis

Immunoblot analysis, and immunoreactive proteins were visualized by an enhanced chemiluminescence (ECL) procedure according to the manufacturer’s protocol. PANC-1 cells (5 × 10^5^ cells/mL) were treated with DMC (3-30 μM) for 24 h at 37 °C. Cell lysates were prepared in 100 μL of lysis buffer (Sigma, Ronkonkoma, NY, USA) containing a protease inhibitor cocktail (Roche, Mannheim, Germany). Insoluble material was removed by centrifugation at 14,000 rpm for 10 min. And then the protein contents in the supernatant were measured using a Bio-Rad DC protein assay kit. The protein extract (50 μg/well) was separated by SDS-PAGE and then transferred onto PVDF membranes (Bio-Rad, Hercules, CA, USA). The membranes were bloked with 5% (*w*/*v*) non-fat dry milk in TBS-T [Tris-buffered saline containing 0.1% (*v*/*v*) Tween-20] at 4 °C overnight and incubated with primary antibodies at room temperature for 1.5 h. The membranes were washed three times with TBS-T, and blotted with secondary antibodies conjugated with horse-radish peroxidase at room temperature for 1.5 h, followed by washing three times in TBST-T. Immunoreactive proteins were visualized by an enhanced chemiluminescence (ECL) procedure according to the manufacturer’s protocol (Santa Cruz Biotechnology, Santa Cruz, CA, USA) and exposed to X ray films. Protein contents were normalized by reprobing the same membrane with anti-β-actin detection; previously used membranes were soaked in stripping buffer (Gene Bio-Application Ltd., Yavne, Israel) at room temperature for 20 min.

### 4.9. Statistical Analysis

All treatments were conducted in triplicate and the results are presented as the mean ± standard deviation (S.D). The statistical significance of all treatment effects was evaluated by Student’s *t*-test with a probability limit for significance of *p* < 0.05, *p* < 0.001.

## Figures and Tables

**Table:** 

	**LL** **(Annexin V−/PI–)**	**LR** **(Annexin V+/PI–)**	**UL** **(Annexin V–/PI+)**	**UR** **(Annexin V+/PI+)**
**Control**	92.4 ± 3.7	2.8 ± 1.0	3.50 ± 0.8	6.1 ± 0.9
**DMC (3 μM)**	85.4 ± 2.3	0.5 ± 0.2	5.5 ± 1.0	8.2 ± 1.4
**DMC (10 μM)**	50.7 ± 4.4	6.2 ± 1.5	12.0 ± 2.5	32.8 ± 2.8
**DMC (30 μM)**	1.0 ± 0.3	0.1 ± 0.1	5.2 ± 0.5	95.1 ± 1.6

## Supplementary Materials

The following are available online, Figure 1: ^1^H NMR spectra of DMC (600 MHz, MeOD). Figure 2: ^13^C NMR of DCM (125 MHz, MeOD).
