# Supplementary figures and images for: The Effects of 2′,4′-Dihydroxy-6′-methoxy-3′,5′- dimethylchalcone from Cleistocalyx operculatus Buds on Human Pancreatic Cancer Cell Lines
Source: Molecules. 2019 Jul 11;24(14):2538. doi: 10.3390/molecules24142538 (PMC6680674; doi:10.3390/molecules24142538)

Figure 1.  $^1\text{H}$  NMR spectra of DMC (600 MHz, MeOD)

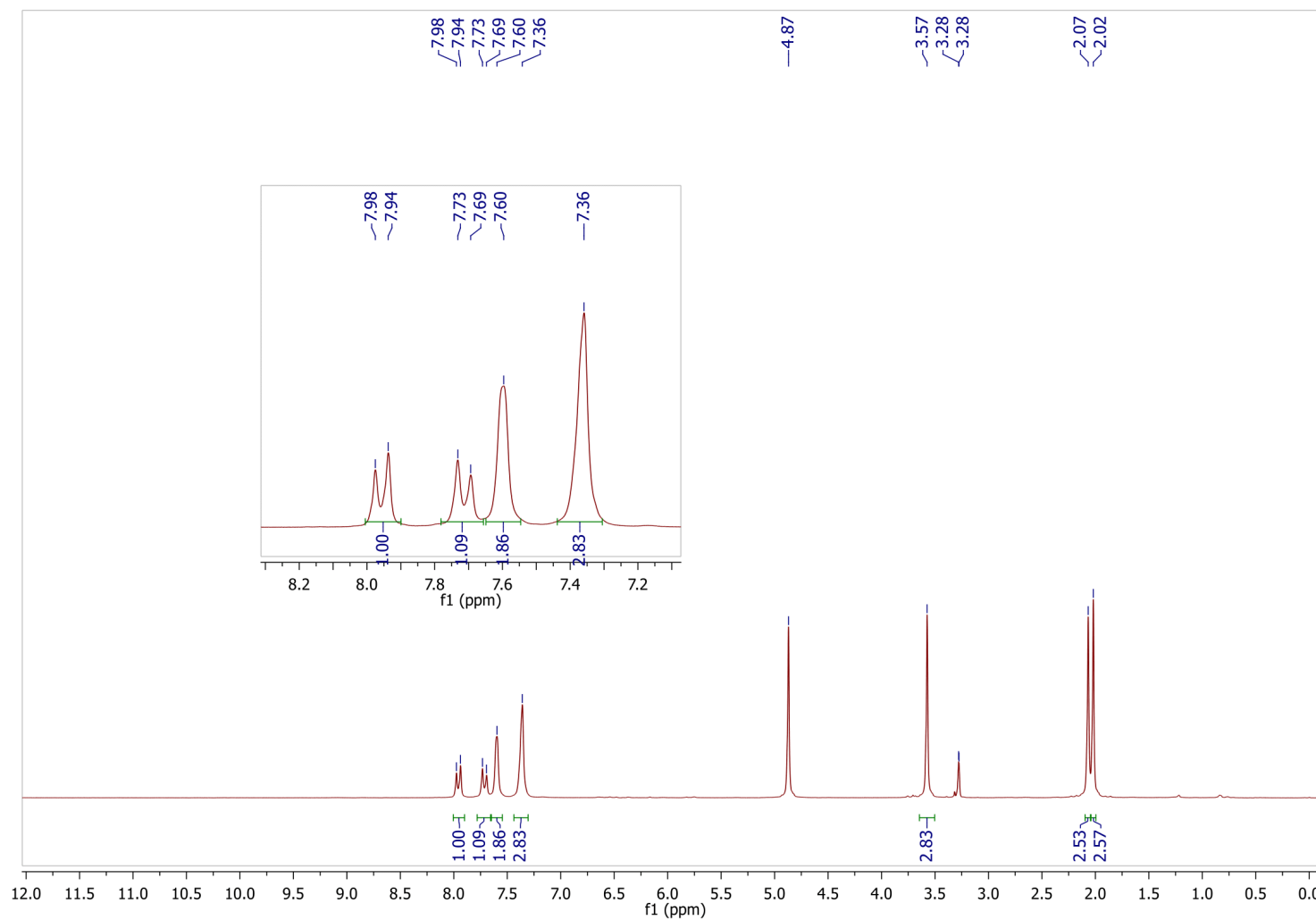

Figure 2.  $^{13}\text{C}$  NMR of DCM (125 MHz, MeOD)

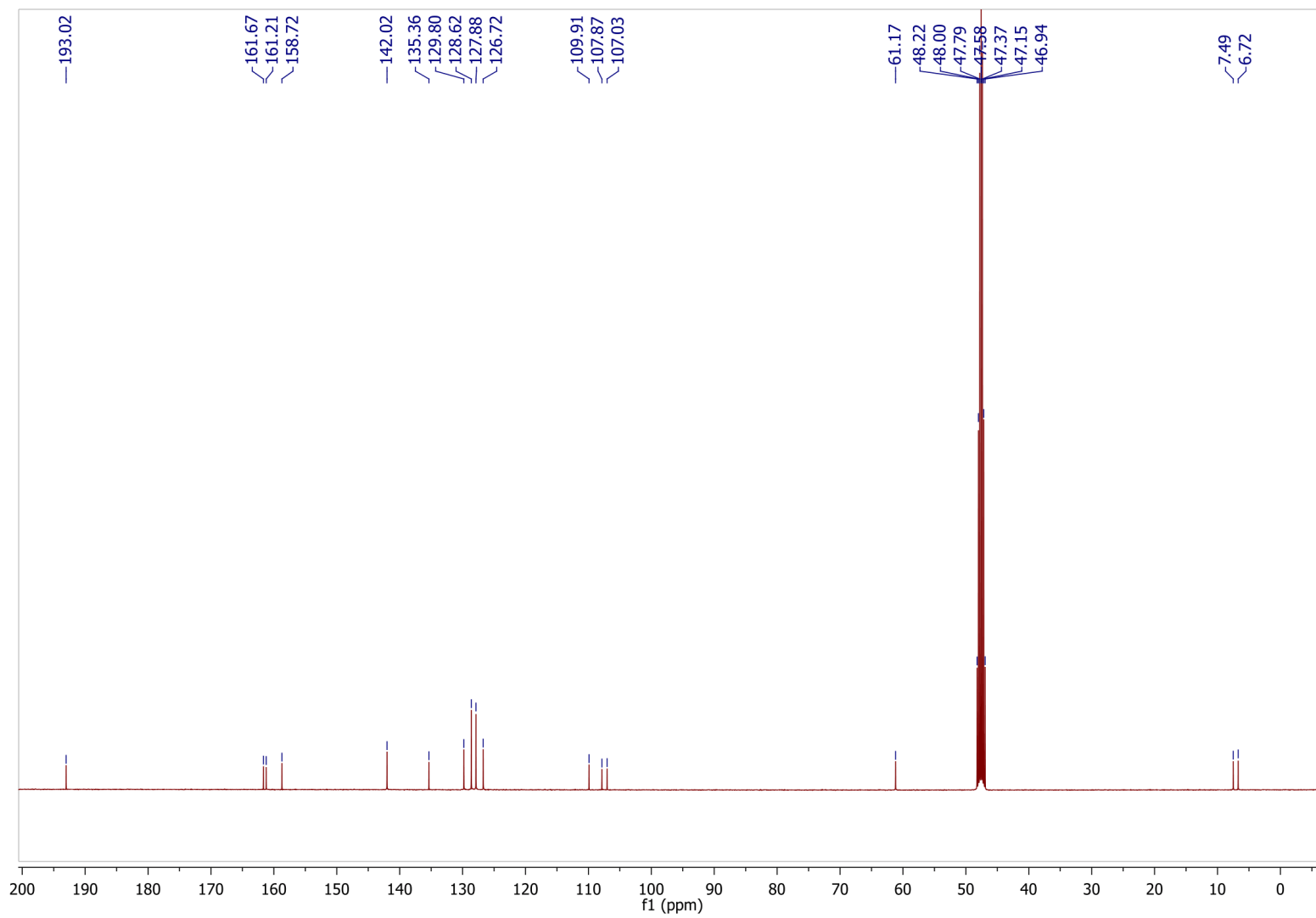

Supplement: Supplementary file 1 [file molecules-24-02538-s001.pdf]
